# Age of onset, treatment response, and survival rates in Dutch Kooiker dogs diagnosed with hereditary polymyositis

**DOI:** 10.3389/fvets.2025.1559040

**Published:** 2025-05-30

**Authors:** Yvet Opmeer, Vanessa Alf, Peter A. J. Leegwater, Hille Fieten, Paul J. J. Mandigers

**Affiliations:** ^1^Expertise Centre of Genetics, Department of Clinical Sciences, Faculty of Veterinary Medicine, Utrecht University, Utrecht, Netherlands; ^2^Neurology Service, Evidensia Referral Hospital Arnhem, Arnhem, Netherlands; ^3^Section of Clinical and Comparative Neuropathology, Centre for Clinical Veterinary Medicine, Ludwig-Maximilians-Universität Munich, Munich, Germany

**Keywords:** myopathy, treatment, oclacitinib, apoquel, vitamin B, coenzyme Q10, L-carnitine, prednisolone

## Abstract

**Introduction:**

Hereditary polymyositis in Kooiker dogs is genetically linked to the deletion of a 39 kb DNA fragment located 10 kb upstream of the interleukin IL21/IL2 locus, exhibiting low penetrance. This study aims to identify prognostic factors for Kooiker dogs that are affected and evaluate three treatments: (1) glucocorticoid monotherapy, (2) glucocorticoids combined with supplements (vitamin B, L-carnitine, and coenzyme Q10), and (3) glucocorticoids combined with supplements and oclacitinib.

**Method:**

This study incorporates both retrospective and prospective designs. A total of 97 Kooiker dogs diagnosed with histopathologically confirmed polymyositis, or those showing clinical signs consistent with polymyositis and homozygous or heterozygous for a 39-kb deletion, were included. Dogs seen before the end of 2006 received only Treatment 1, those identified between 2007 and 2015 received Treatment 2, and those seen after 2015 received Treatment 3. A dataset was created that included sex, neuter status, age of onset (AoO), the 39 kb deletion, clinical signs, treatment, creatinine kinase (CK) activity at diagnosis and during follow-up visits, owner-reported improvement during treatment, cause of death, and date of death. Descriptive statistics and Cox proportional hazards survival analysis were conducted to examine factors influencing age of onset, treatment response, and survival.

**Results:**

The 39 kb deletion showed no association with clinical signs or creatine kinase (CK) activity at diagnosis. However, homozygous Kooiker dogs with the 39 kb deletion had a lower AoO. A significant decrease in CK activity was observed in 18 dogs during the first recheck after treatment, and most owners reported clinical improvement following the start of treatment. The type of treatment influenced survival time, with dogs treated with glucocorticoids, supplements, and oclacitinib having the longest survival.

**Discussion:**

A treatment regimen that included glucocorticoids, supplements, and oclacitinib was the most effective in this study, although it was not statistically significant.

## Introduction

In 2023, hereditary polymyositis was described in ‘Het Nederlandse Kooikerhondje’, hereafter referred to as the Kooiker dog (1). The Kooiker dog, originally used for catching ducks in a decoy, is one of the nine Dutch breeds with a history that dates back to the 16th century (2, 3). After losing its purpose, the breed faced near extinction. However, after the Second World War, approximately 10 Kooiker dogs were used to help re-establish this breed. Using such a limited pool for breed restoration raises the risk of genetic disorders (4). The breed has been affected by several hereditary diseases, including hereditary myelopathy (5, 6), Von Willebrand disease (7), and polymyositis (1, 8). Although originally a Dutch breed, it is now found throughout Europe, the USA, and Japan (9), and the total population size is estimated to be up to 38,000 dogs, of which 15,000 are living dogs (10).

The clinical signs of polymyositis in this breed can include locomotor problems, dysphagia, or a combination of both and are described in detail by Opmeer et al. (1). The majority of dogs simultaneously exhibit several clinical signs. Examples of locomotor issues are the inability to walk long distances as they become paretic, difficulty getting up, a stiff gait, and walking on eggshells. An earlier study demonstrated that the predominant clinical signs were difficulty walking in 92% of all Kooiker dogs and a stiff gait in 51%. Not all Kooiker dogs with polymyositis exhibit clear clinical signs, especially in the early stages of the disease; these signs can be vague and non-specific. However, the longer the dogs suffer from this disease, the more they deteriorate as it progresses. Up to 40% of Kooiker dogs had difficulty drinking and/or eating. Sialorrhea is observed while eating and drinking in a quarter of all dogs. Nearly half of all dogs developed anorexia. Dyspnea, panting, and coughing were present in approximately 20% of all dogs.

The histopathology of affected Kooiker dogs has been described in detail in two earlier studies (1, 8). Affected Kooiker dogs exhibit moderate to marked, chronic-active, diffuse, interstitial, and myofiber-directed lymphohistiocytic myositis, typically with a low and variable number of eosinophils, neutrophils, and plasma cells. In several biopsies, degeneration (necrosis), regeneration, atrophy, and fibrosis were observed (1, 8). Recently, the results of a genome-wide association study and next-generation sequencing of polymyositis in the Dutch Kooiker dog have been published (11). The deletion of a 39 kb DNA fragment located 10 kb upstream of the interleukin *IL21*/*IL2* locus is associated with the development of polymyositis (11, 12). Leukocytes from untreated affected dogs, which are homozygous for the deletion, overexpress *IL21* and *IL2* upon mitogen stimulation (11, 12). The frequency of the deletion allele was 0.81 in the cases and 0.25 in a random sample of the Kooiker dog, indicating that the penetrance of the disease phenotype is estimated to be 10–20% for homozygous dogs and 0.5–2% for heterozygous dogs carrying the deletion. Therefore, homozygous and heterozygous dogs are at risk of being affected, with the risk being significantly higher for homozygous dogs.

Opmeer et al. (1) demonstrated that CK activity is elevated (107 dogs; 2056 ± 1,601 U/L (mean ± SD); range 179 to 8,466 U/L) in almost all Kooiker dogs when they are affected. Measuring CK activity can help identify affected dogs when clinical signs are observed. If CK activity is high, it can be paired with the available DNA test (11).

The outcome of polymyositis in the Kooiker dog is often poor (1). Affected Kooiker dogs have been recognised and referred for diagnosis and treatment since 1990. Initially, glucocorticoids served as the standard therapy for immune-mediated diseases, such as polymyositis (13, 14). In 2006, the therapeutic regimen for polymyositis was empirically enhanced to include L-carnitine, coenzyme Q10, and vitamins B6 and B12. L-carnitine is used as a supplement in human patients with myopathies to support muscle function (15, 16), while coenzyme Q10 can act as a non-specific stimulant for the immune system (17). However, in human medicine, some guidelines advise its use in mitochondrial myopathy and not just any myopathy (18). It may, however, improve symptoms associated with statin-related muscle signs (19). Vitamin B complex, particularly vitamin B_6_ and B_12_, is crucial for various neuronal metabolic processes, including axonal transport, neurotransmitter synthesis, and antioxidant mechanisms, which are essential for managing inflammatory responses (20–22). With the discovery of the 39 kb deletion and the upregulation of *IL2* and *IL21* in leukocytes of Kooiker dogs with the 39 kb deletion (11), the Janus Kinase (JAK) inhibitor oclacitinib^1^ was added to the treatment in 2015. Oclacitinib inhibits *IL2* signalling (23) and depletes CD8 + T cells (24).

This study aims to identify prognostic factors in affected Kooiker dogs and to evaluate three treatment regimens: (1) glucocorticoid monotherapy, (2) glucocorticoids combined with supplements (vitamin B, L-carnitine, and coenzyme Q10), and (3) glucocorticoids combined with supplements and oclacitinib.

## Materials and methods

### Dogs

All Kooiker dogs included in the study were purebred Kooiker dogs exhibiting clinical signs indicative of polymyositis, supported by a confirmed histopathological diagnosis of polymyositis (1), or clinical signs consistent with polymyositis, either homozygous or heterozygous for the 39 kb deletion, but without histopathology (11, 12). All Kooiker dogs were referred to the last author (PJJM) for diagnosis and treatment. These dogs primarily originated from the Netherlands, Belgium, Luxembourg, or Germany. If it was a Kooiker dog from another country, the last author (PJJM) was consulted by email for advice on diagnosis and treatment. The dataset included date of birth, sex, neuter status, kennel club registration number, country of origin, AoO, clinical signs, 39 kb mutation status, creatinine kinase (CK) activity at the time of diagnosis and during treatment (reference value 10–200 U/L), therapy, owner-reported response to treatment, date, and cause of death. The dog was excluded from this study if the dataset could not be completed.

All dogs were privately owned. Before the study began, informed consent was obtained from the owner. Approval from the animal welfare body of Utrecht University was requested but waived, as it concerned registered treatments for dogs. We adhered to the requirements for compliance with good laboratory practice for veterinary medicinal products, as set out in Annex II of Regulation (EU) 2023/183 of 23 November 2022, amending Regulation (EU) 2019/6 of the European Parliament and of the Council.

### Treatment protocols

Three distinct treatment protocols were applied. The first treatment utilized before 2005 was a monotherapy with glucocorticoids (TG1). In 2006, this therapy was combined with supplements (vitamin B, L-carnitine, and coenzyme Q10) (TG2). Following the discovery of the 39 kb deletion in 2015, the treatment with glucocorticosteroids and supplements was further combined with oclacitinib (TG3). The initial dose of glucocorticoid was 1 mg/kg body weight of prednisolone, and the dosage was adjusted based on clinical efficacy and, if feasible, the CK activity. A reference value for CK activity was set at 10–200 U/L (1). The dosage of oclacitinib was according to the manufacturer’s recommendations: 0.4–0.6 mg/kg twice daily for 14 days, followed by the same dose once daily. For the supplements, owners were instructed to administer 500 mg of L-carnitine twice daily and 60 mg of coenzyme Q10 once daily for Kooiker dogs weighing approximately 6–9 kg, increased to 100 mg for larger Kooiker dogs weighing approximately 9 to 14 kg. In addition, dogs received 1.25–2.5 mg of vitamin B6 and 3–12.5 μg of vitamin B12 daily.

### Owner-reported response to treatment

At each consultation, whether in person, by telephone, or via email, the owners were asked to score their dog’s ability to walk, drink, and eat using a visual analogue scale from 0 to 100. This score was compared to the situation before treatment, during treatment, and against the situation before the dog fell ill. Owners could score their dog as follows: (1) clear improvement in clinical signs to normal or nearly normal conditions if the QoL score was higher than 75%; (2) moderate response if the QoL score ranged from 25 to 75%; (3) no response if it fell between 0 and 25%; and (4) deterioration if the QoL score was worse than before the start of treatment.

### Statistics

The results were analysed using R version 4.4.2, with the ggplot2, survival, and survminer packages. Descriptive statistics summarised the data in the dataset. Data that were not normally distributed were presented as mean and range. All variables were tested for normal distribution using the Kolmogorov–Smirnov test. Except for sex, the variables were not normally distributed, so a Mann–Whitney test was used to analyse the relationship between 39 Kb deletion status, age of onset (AoO), and CK-activity. A Kruskal–Wallis test was conducted for clinical presentation, owner-reported response to treatment, 39 Kb deletion, AoO, and CK-activity. A Pearson’s Chi-squared test was used to examine the distribution of 39 Kb deletion among different sexes.

Hazard ratios with 95% confidence intervals were estimated using Cox-proportional hazard regression analysis to investigate factors associated with the risk of dying of polymyositis. An event was defined as death due to polymyositis, dogs that were alive at the time of analysis, died of another cause, or were lost to follow-up were censored. Factors that were investigated included the age of onset of clinical signs, 39 kb deletion status, sex, CK level at the time of diagnosis, clinical signs at presentation, and therapy.

## Results

### Description of dogs

A total of 97 out of 135 Kooiker dogs suffering from clinical signs suggestive of polymyositis were included in this study. Thirty-eight Kooiker dogs were excluded as the dataset could not be completed. Histopathology confirmed polymyositis in 81 Kooiker dogs. Histopathology was absent in the other 16 dogs, but they exhibited clear clinical signs suggestive of polymyositis and were either homozygous or heterozygous for the 39 kb deletion (1, 8, 11). The status of the 39 Kb deletion was established in 81 Kooiker dogs. Fifty-eight of these 81 Kooiker dogs (72%) were homozygous for the 39 kb deletion, while 23 were heterozygous (28%). DNA samples were missing for 16 Kooiker dogs, but these dogs had confirmatory histopathology. For all 97 dogs, CK activity was measured at the time of diagnosis. The cohort included 41 females (29 intact and 12 neutered) and 56 males (44 intact and 12 neutered). The majority of the included dogs originated from the Netherlands (*n* = 63), with 31 from other European countries, two from North America, and one from Japan. All Kooiker dogs descended from the original founder dogs, as published earlier (2, 5).

### Clinical presentation

At the time of diagnosis, 42 Kooiker dogs (43.2%) exhibited only locomotion issues, 9 dogs (9.3%) had only dysphagia, and 46 dogs (47.4%) had a combination of locomotion problems and dysphagia. All these dogs have been described in detail by Opmeer et al. (1).

### Age of onset

The median AoO in the 97 dogs was 3.5 years (range 1.0–10.4 years). There was no statistical difference between AoO and sex (Mann–Whitney test, *p* = 0.423) and between AoO and clinical presentation (Kruskal–Wallis, *p* = 0.343; Table 1). There appears to be an effect of neutering status, with a higher AoO for neutered bitches and sires (Kruskal–Wallis, *p* = 0.018).

**Table 1:** AoO and CK activities were assessed for the three variants of clinical presentations.

Clinical signs	AoO in years	CK activity (U/l)
Locomotion	4.3 ± 2.4	1,527; range 254–6,676
Dysphagia	3.2 ± 1.8	1,083; range 330–4,763
Combination	3.8 ± 1.7	1,500; range 179–7,286
	*p* = 0.343	*p* = 0.337

No statistically significant differences were found among these three clinical presentations.

### Creatinine kinase activity

The median CK activity for the total group of 97 dogs was 1,442 U/L (range 179–7,286 U/L). Using an upper reference level for CK activity of 200 U/L, one dog had normal CK values. There was no statistically significant difference between clinical presentation and CK activity (Kruskal–Wallis; *p* = 0.337) at the time of diagnosis (Table 1).

### 39 kb deletion

**Figure 1:**
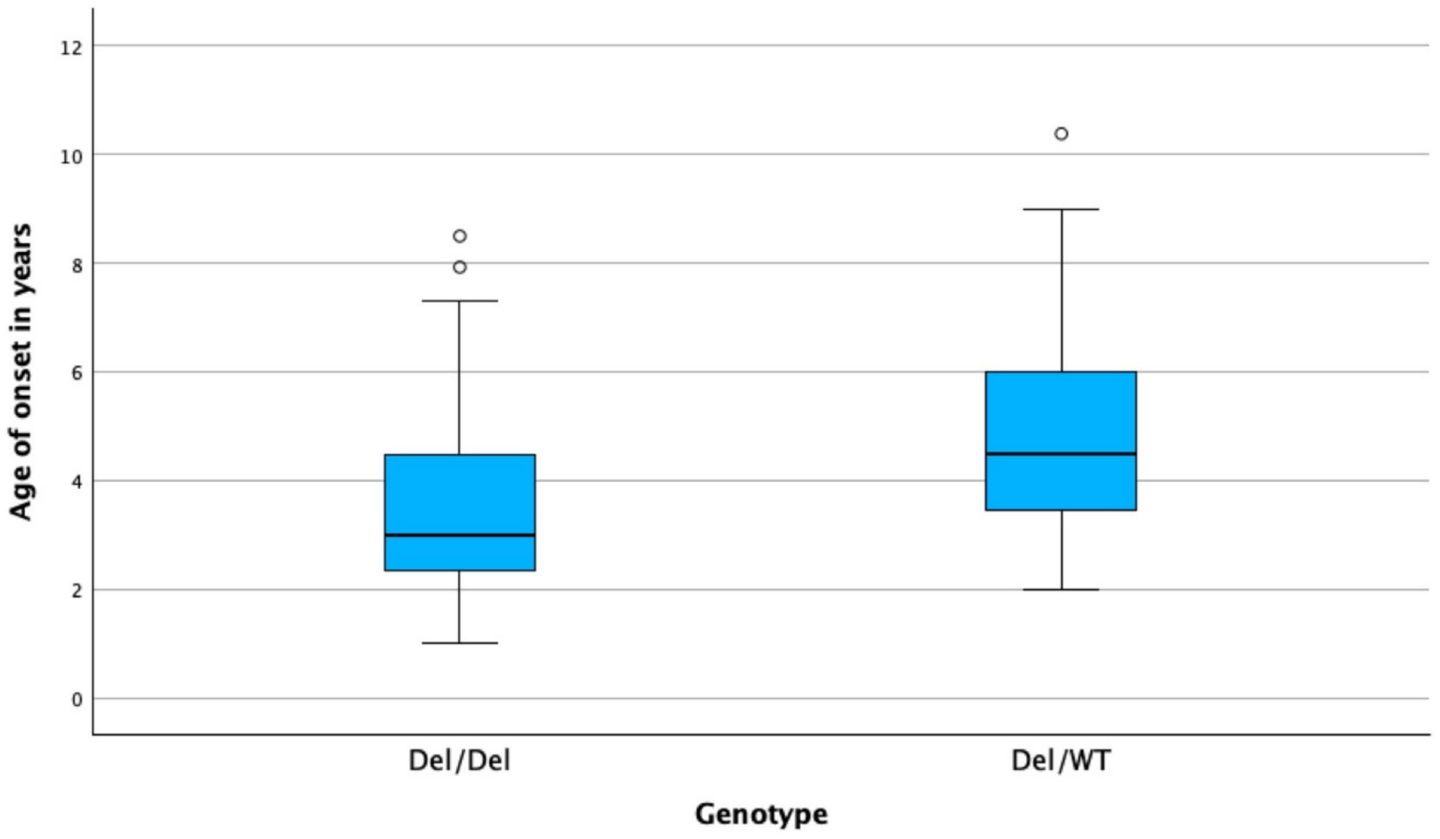
Box plot illustrating AoO for the two genotypes: homozygous (Del/Del) and heterozygous (Del/WT). The difference is statistically significant (*p* = 0.011).

There was no significant relationship between the type of clinical presentation and the 39 kb deletion status (Mann–Whitney test, *p* = 0.697), nor was there a significant association for CK activity (Mann–Whitney test, *p* = 0.09). Additionally, there was no difference in the distribution of the 39 kb deletion status among the different categories of sex (chi-squared; *p*-value = 0.14). The AoO for 39 kb deletion status had a median age of 3 years (range 1–8.5) for the homozygous dogs (Del/Del) and 4.5 years (range 2–10.4) for the heterozygous dogs (Del/WT). This difference was statistically significant (Mann–Whitney test, *p* = 0.011) (see Figure 1).

### Treatment—treatment groups

Fifty-three dogs received only prednisolone (TG1, 55%), 24 dogs received prednisolone plus supplements (TG2, 25%), and 15 dogs were given prednisolone, supplements, and oclacitinib (TG3, 15%). Additionally, three Kooiker dogs that did not receive any treatment died of polymyositis at the ages of 4.1, 3.0, and 3.5 years, following respective disease periods of 9.3, 8.8, and 0.1 months. One dog treated with prednisolone and cyclosporin died of polymyositis at 2.9 years of age after 11.3 months of illness. Another dog, treated with prednisolone, azathioprine, and chlorambucil, died at 7.4 years of age after a disease period of 29.1 months. As a consequence of the medication, this dog developed bloody diarrhoea. These five Kooiker dogs were excluded from the analysis and were not included in any of the three treatment groups.

### Owner-reported response to treatment

The owner-reported treatment responses were available for 43 dogs. After a median follow-up of 6.4 months (range 1.6–45.4), 36 owners noted a clear improvement in clinical signs, achieving normal or nearly normal conditions (VAS higher than 75%). Six reported no response (VAS between 0 and 25%), and one owner indicated a deterioration of the clinical signs (VAS lower than 0%). There was no difference in how the owners perceived their dog’s response to treatment and the treatment group (Kruskal–Wallis test, *p* = 0.6). All owners reported a similar response to the treatment. Additionally, there was no difference in the 39 kb mutation status: both homozygous and heterozygous dogs responded comparably to treatment. However, owners of homozygous dogs reported a slightly better response than those with heterozygous dogs (Mann–Whitney test, *p* = 0.07).

### CK activity during treatment

After a median time of 6.7 months (range 2.5–45.4), CK activity was measured in 18 dogs. In these dogs, median CK activity decreased significantly from 1,337 U/L (range 450–4,692 U/L) to 222 U/L (range 40–1,122 U/L), with normalisation observed in eight dogs (reference range <200 U/L). Across the three treatment groups, median CK activity decreased at follow-up was: 792 U/L (range: 300–1,122) in TG1, a median of 147 U/L (range: 91–669) in TG2, and a median of 210 U/L (range: 40–323) in TG3. The difference in CK activity between these groups is statistically significant (ANOVA test, *p* = 0.02).

### Survival analysis

At the time of analysis, six Kooiker dogs were still alive. Of the 91 dogs that died during the study period, 84 died due to polymyositis, and one dog died of old age. Out of the 84 dogs that succumbed to polymyositis, four passed away during their illness, whereas 80 were euthanised. Reasons for euthanasia included severe clinical signs such as a complete inability to walk due to significant muscle loss, difficulty drinking or eating, or exhibiting severe respiratory distress, sometimes resulting from aspiration pneumonia. Twelve dogs were excluded from the survival analysis: five Kooiker dogs mentioned earlier did not receive treatment or were treated differently from those in one of the three treatment groups, and the other seven dogs died due to the development of neoplasia. Among these, three dogs succumbed to unspecified lung tumours, one to haemangiosarcoma, one to lymphoma, and two littermates to mast cell tumours.

The overall median survival—defined as the time at which 50% of the Kooiker dogs had not yet died from polymyositis—was 9.8 months.

**Figure 2:**
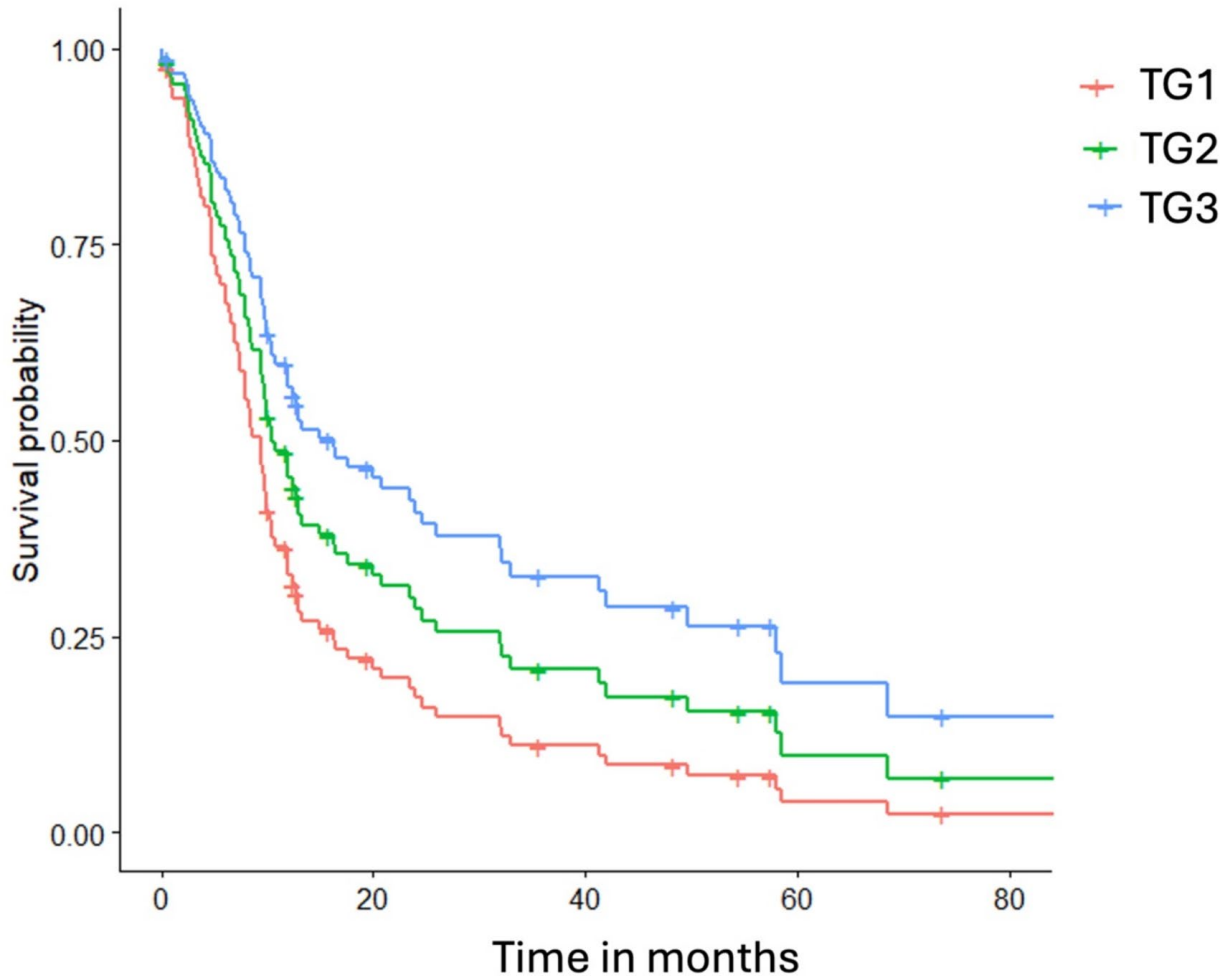
illustrates the cumulative hazard probability curves for death due to polymyositis across different therapy groups, adjusted for the mean age of onset. TG1 represents glucocorticoids only, TG2 indicates a glucocorticoid combined with supplements, and TG3 includes TG2 plus oclacitinib.

The Cox proportional hazards analysis indicated that both the treatment group and AoO influenced the hazard ratio for mortality due to polymyositis. After adjusting for the AoO, the hazard ratio for therapy group 2 compared to therapy group 1 (reference category) was 0.81 (95% CI 0.48–1.37), and for therapy group 3 compared to therapy group 1, it was 0.46 (95% CI 0.21–0.97). The hazard ratio for the age of onset in this multivariable model was 1.09 (95% CI 0.98–1.21) (Figure 2).

## Discussion

This study outlines three treatment options and the outcomes in Kooiker dogs affected by breed-related conditions, such as polymyositis. Additionally, we aimed to determine whether it is possible to identify prognostic factors. Neither age, sex, clinical presentation, nor CK activity was an indicative prognostic factor for survival outcomes. Kooiker dogs that were neutered, both males and females, had a statistically significant higher age of onset. This contrasts with other reports indicating that neutering increases the risk of developing an immune-mediated disease. In these dogs, we observed the opposite.

Interestingly, Kooiker dogs that were homozygous for the 39 kb deletion experienced an earlier AoO for clinical polymyositis. Although this finding is a predictive factor, it cannot be considered a prognostic survival outcome factor. Regardless of genotype, both homozygous and heterozygous Kooiker dogs exhibit similarly poor outcomes, but homozygous Kooiker dogs appear to develop polymyositis at a younger age.

Polymyositis in Kooiker dogs is an immune-mediated myopathy that has traditionally been treated with glucocorticoids, which inhibit T-cell activity and induce T-cell apoptosis (13, 14). The pathophysiology of canine polymyositis is generally characterised by the infiltration of mononuclear inflammatory cells, predominantly CD8 + T-cells, into striated skeletal muscles (8). Therefore, using glucocorticoids is a logical therapeutic approach for affected dogs. The first Kooiker dogs identified with this disease, dating back to 1994, were treated solely with prednisolone, and in 2005, supplements were added. Among these, L-carnitine is used in patients with myopathies (15, 16). L-carnitine is believed to alleviate muscle injury, reduce markers of cellular damage, and decrease free radical formation (16). By increasing serum and muscle L-carnitine levels, the supplement improves blood flow and oxygen delivery to the muscle tissue through enhanced endothelial function, thereby mitigating hypoxia-induced cellular and biochemical disruptions (16). Contrary to the conclusions of Brevetti et al. (15), the authors are unaware of any specific studies investigating the efficacy of L-carnitine in treating polymyositis. Some reports suggest that coenzyme Q10 can function as a non-specific immune stimulant (17, 25). A placebo-controlled study involving human patients with multiple sclerosis demonstrated that coenzyme Q10 supplementation (25) significantly decreased TNF-*α* and IL-6 levels compared to the placebo group. However, this does not necessarily indicate effectiveness in canine polymyositis. Nonetheless, coenzyme Q10 is occasionally utilised as a part of a treatment protocol for patients with mitochondrial myopathy (18, 26, 27). Similar reports have been published regarding the potential therapeutic benefits of various B vitamins, although none specifically address their application to myopathies or polymyositis. However, none of the reports provide strong scientific evidence for their efficacy. It should be noted that a Cochrane review of mitochondrial therapies found little evidence supporting the use of any vitamin or cofactor (19).

After the identification of the associate mutation in 2015, oclacitinib was added to the treatment regimen. The 39 kb deletion disrupts the regulatory mechanisms controlling IL2 and IL21 expressions (11). IL21 signalling is mediated through the Janus Kinase (JAK)-signal transducer and activator of transcription (STAT)-signalling, also called JAK/STAT pathway (5). The overexpression of IL21 in affected dogs is associated with activating the JAK1 and JAK3 receptors, suggesting that targeting this signalling cascade with JAK inhibitors may provide a novel therapeutic approach for polymyositis in the Dutch Kooiker dog. Oclacitinib has been shown to selectively inhibit JAK1-dependent cytokines and IL-2 signalling (23) and deplete CD8 + T cells (24). The Cox proportional hazards analysis indicated that both the treatment group and AoO influenced the hazard ratio for death due to polymyositis. We accounted for AoO, as older dogs typically have a shorter survival time following disease onset, irrespective of treatment response. Based on the analysis, TG2 and TG3 are superior to TG1. There was no significant difference between TG2 and TG3, although dogs in TG3 exhibited lower CK activity at their first control and demonstrated a slightly higher survival probability. There was no statistically significant difference between the owner-reported response to treatment and the treatment group. However, if CK activity reflects clinical improvement, the data suggest that treatment including oclacitinib may be superior. In this study, we could not determine how quickly the prednisolone dose was reduced. A future research question is whether the use of oclacitinib can reduce prednisolone use and decrease glucocorticoid-related side effects.

Seven dogs (7.6%) developed neoplasia during the course of their illness, a prevalence lower than that reported for the general dog population (28), and the 11 to 21% prevalence reported by the Kooiker dog breed club (9, 10). The association between neoplasia and polymyositis observed in Boxers (29) does not appear to be present in this breed.

This study has limitations. It is partially retrospective, with the first cases dating back to 1994. Cases seen before 2005 were retrieved retrospectively, and sadly, the registration of clinical data was not always complete. Additionally, data were collected from various owners and veterinarians across multiple countries, each using their own administration systems. Although standardised instructions for diagnosing polymyositis were provided in 2010, some information may still have been overlooked. Furthermore, the clinical efficacy was established based on the owner’s subjective reports, as a clear, objective measure for assessing treatment efficacy was absent. In several cases, this feedback was insufficient, leading to the exclusion of several dogs from this part of the analysis. While CK activity could serve as a marker, it was not measured consistently at standardised time points.

Despite the limitations, we formulated some recommendations based on the retrieved data. Kooiker dogs homozygous for the 39 kb deletion exhibited an earlier age of onset (AoO). Additionally, neutered Kooiker dogs also showed a higher AoO. The recommendation is to monitor Kooiker dogs carrying the 39 kb deletion, regardless of being homozygous or heterozygous, at regular intervals to facilitate earlier diagnosis. This study does not provide a definitive conclusion on whether the treatment improves prognosis. However, there are indications that the combination of a glucocorticoid, supplements, and oclacitinib may be the most beneficial treatment regimen and could potentially increase life expectancy. Further studies are needed to confirm these findings and to improve treatment strategies for Kooiker dogs with polymyositis.

## Conclusion

Kooiker dogs homozygous for the 39 kb deletion have an earlier AoO. Neutered Kooiker dogs have a higher AoO. Although not statistically significant, treatment with glucocorticoids, supplements, and oclacitinib seems to be the most beneficial for Kooiker dogs affected by polymyositis. Future studies are needed to improve the treatment of affected Kooiker dogs.

## Data Availability

The raw data supporting the conclusions of this article will be made available by the authors, without undue reservation.

## Footnotes

^1^Oclacitinib (Apoquel®) Zoetis - https://www.zoetisus.com/products/dogs/apoquel
